# Hybridization and amplification rate correction for affymetrix SNP arrays

**DOI:** 10.1186/1755-8794-5-24

**Published:** 2012-06-12

**Authors:** Quan Wang, Peichao Peng, Minping Qian, Lin Wan, Minghua Deng

**Affiliations:** 1 Center for Theoretical Biology, Peking University, Beijing, 100871, People's Republic of China; 2 LMAM, School of Mathematical Sciences, Peking University, Beijing, 100871, People's Republic of China; 3 Molecular and Computational Biology Program, University of Southern California, Los Angeles, CA, USA; 4 National Center for Mathematics and Interdisciplinary Sciences, and the Key Laboratory of Systems and Control, Academy of Mathematics and Systems Science, Chinese Academy of Sciences, Beijing, 100190, People's Republic of China; 5 Center for Statistical Science, Peking University, Beijing, 100871, People's Republic of China

**Keywords:** SNP array, Copy number variation (CNV), Cross-hybridization, Genomic waves

## Abstract

**Background:**

Copy number variation (CNV) is essential to understand the pathology of many complex diseases at the DNA level. Affymetrix SNP arrays, which are widely used for CNV studies, significantly depend on accurate copy number (CN) estimation. Nevertheless, CN estimation may be biased by several factors, including cross-hybridization and training sample batch, as well as genomic waves of intensities induced by sequence-dependent hybridization rate and amplification efficiency. Since many available algorithms only address one or two of the three factors, a high false discovery rate (FDR) often results when identifying CNV. Therefore, we have developed a new CNV detection pipeline which is based on hybridization and amplification rate correction (CNVhac).

**Methods:**

CNVhac first estimates the allelic concentrations (ACs) of target sequences by using the sample independent parameters trained through physicochemical hybridization law. Then the raw CN is estimated by taking the ratio of AC to the corresponding average AC from a reference sample set for one specific site. Finally, a hidden Markov model (HMM) segmentation process is implemented to detect CNV regions.

**Results:**

Based on public HapMap data, the results show that CNVhac effectively smoothes the genomic waves and facilitates more accurate raw CN estimates compared to other methods. Moreover, CNVhac alleviates, to a certain extent, the sample dependence of inference and makes CNV calling with appreciable low FDRs.

**Conclusion:**

CNVhac is an effective approach to address the common difficulties in SNP array analysis, and the working principles of CNVhac can be easily extended to other platforms.

## Background

Copy number variations (CNVs) play an essential role in facilitating human diseases susceptibility [1,2] and have been shown to be one potential source of missing heritability of complex diseases [3]. Together with genome-wide association studies (GWAS), CNVs are predicted to be compelling in deciphering the pathology of human diseases [4]. SNP arrays have been widely used for CNV studies, and tremendous data have been generated [5-7]. Although high throughput sequencing technologies are emerging and have been applied to genetic variation (including CNV) studies, the cost of a sequencing-based approach is still higher than traditional SNP arrays, especially in library construction [8]. In addition, various studies have shown that the sequencing data are not sensitive to breakpoint detection [9-11]. Moreover, sequencing technologies have poor mutation detection capability when the sequencing coverage (read depth) is relatively low [12]. Thus, at their current stage of development, we believe that sequencing technologies are complementary, not substitute, tools of SNP arrays. Therefore, in this article, we aim to develop a new and more accurate CNV detection pipeline that avoids the common difficulties in SNP array analysis.

High quality CNV calls for accurate estimation of raw copy numbers and requires that statistical models be optimized [6]. Although many methods have been developed for CNV calling from array-based data [7,13-16], their accuracies are still far from satisfactory by the high incidence of false discovery rates (FDRs) [5,17-19]. The high FDRs of these methods mainly arise from (1) cross-hybridization of probes [20], (2) genomic waves of intensities [21-23] and (3) sample dependence of outputs [24-26].

Cross-hybridization between probes and off-target sequences is a longstanding problem in microarray analysis [27-30]. Therefore, most previous methods have typically ignored cross-hybridization and focused on taking mean or median intensities of probes as the estimated raw CNs [15,31]. However, such estimated CNs hardly reflect the true allelic concentrations (ACs) of target sequences, and some studies [6,7,20] have shown that cross-hybridization, if not considered, can lead to large bias. To circumvent this problem, one prior investigation used PICR (probe intensity composite representation) to model the hybridization and cross-hybridization based on the underlying physicochemical principle of DNA/DNA duplex formation in array experiments, and then removed the effect of cross-hybridization and accurately estimated AC at a given SNP site through a statistical method [20]. Other similar models were also reported [28,32].

In addition to cross-hybridization, Maris et al. have stated that “whole-genome microarrays with large-insert clones designed to determine DNA copy number often show variation in hybridization intensity that is related to the genomic position of the clones.” [22] These ‘genomic waves’ have been observed in SNP arrays [21-23]. Genomic waves are shown to be correlated with GC-content [21,23] and may stem from the amplification of DNA fragments [33]. In the preprocessing of arrays, DNA samples are first digested with restriction enzymes, such as Nsp, and then ligated with adapters before amplification. However, owing to differences in amplification efficiencies of fragments, the PCR procedure can bring in artifacts which may give rise to genomic waves [33]. Presence of the waves will hamper detection of aberrations [23] and introduce hundreds of potentially confounding CNV artifacts that can obscure bona fide variants [33]. To solve this difficulty, a computational approach via fitting regression models with GC-content included as a predictor variable was proposed by [22], and this approach have improved the accuracy of CNV detection.

Finally, it has long been known that different sample batches can lead to inconsistent results, even if data are collected by the same lab [24-26]. Owing to this effect, statistical power in meta-analysis of multiple samples may be significantly reduced [34]. Almost all existing algorithms require multiple samples for training because of the numerous parameters, while different training sample batches can lead to different parameter estimation. The inconsistencies may be incurred by this sample-dependent parameter estimation. The effect has also been shown to be correlated with differences in batch sizes and the extent of homogeneity of samples in each batch. Hence, samples with high homogeneity are suggested to be placed into the same training batch [26]. Several other methods to adjust this batch effect have also been proposed, such as [25,35,36].

To the best of our knowledge, existing methods only address one or two of the three factors discussed above. In this study, we developed a novel CNV detection pipeline based on hybridization and amplification rate correction (CNVhac^a^) to accurately detect CNVs for Affymetrix SNP array. In contrast to previous methods, CNVhac takes into account all three factors by proper modeling of cross-hybridization, smoothing genomic waves and alleviating sample batch dependence of parameter estimation, thus significantly improving the accuracy of CNV detection. Starting from dozens of basic constants concerning binding affinity, which can be well trained from one single array and are quite stable between arrays, CNVhac is able to get the binding affinity between all probes and sequences without suffering from sample batch dependence. Then CNVhac applies the PICR method [20] to address the effect of cross-hybridization. Finally, since we have found that the relative amplification efficiencies between different fragments are fairly stable from one array to another, a simple adjustment approach is proposed to smooth the genomic waves. Based on the accurate raw CN estimates, a hidden Markov model (HMM) is also proposed to detect breakpoints along the genome. The implementation of CNVhac with public datasets shows that our method does enhance the power of both raw CN estimation and CNV calling.

## Methods

### Dataset

Dataset I. ‘The International HapMap project’ [37] mapped 270 samples (30 YRI trios, 30 CEU trios, 45 CHB and 45 JPT individuals) to Affymetrix SNP 6.0 array to identify and catalog genetic similarities and variants in human beings. The raw SNP 6.0 dataset (http://www.affymetrix.com/support/technical/sample_data/genomewide_snp6_data.affx) is applied in this paper.

Dataset II. Conrad et al. recently used the ultra-high-resolution NimbleGen tiling arrays (42 M probes) to identify CNVs for HapMap samples [38]. The identified CNVs were then filtered by two other technologies (Agilent and Illumina). Finally, over 5000 regions that were cross-platform verified as CNV in at least one of the HapMap individuals of dataset I were selected [38] and referenced as benchmark in this article to assess the power of CNV calling in comparison with other algorithms. We have not performed any experimental research by ourselves, and both dataset I and II are downloaded from public databases. Therefore, there is no ethical approval problem in this study.

### Estimation of raw CNs

The problems usually confronted in the estimation of raw CNs are discussed in the background section. Array intensities not only rely on ACs of target sequences, but also probe binding affinities. Based on [20], we model hybridization and cross-hybridization with dozens of probe-independent parameters, which can be accurately estimated from single array and are consistent between arrays [39]. Another simple adjustment is proposed to calibrate the various amplification efficiencies.

#### Modeling hybridization and cross-hybridization

Considering one probe in a certain SNP probeset, we have the basic model [39,40]:

(1) $$I=I_{s}+I_{\mathrm{bg}}+\varepsilon \text{,}$$

where *I, I*_*s*_ and *I*_*bg*_ stand respectively for probe intensity, specific hybridization intensity caused by target sequences and background nonspecific binding intensity, and *ϵ* is the measurement error. *I*_*s*_ has been further modeled by Langmuir-like adsorption principle, and Equation (1) can be rewritten as:

(2) $$I=I_{s}+I_{\mathrm{bg}}+\varepsilon =\frac{N}{1+\exp\left(E\right)}+I_{\mathrm{bg}}+\varepsilon \text{,}$$

where *N* is AC of the target sequences, and *E* denotes specific binding free energy which can be modeled by position-dependent nearest-neighbor (PDNN) [39,40]:

(3) $$E=\sum_{i=1}^{24}\omega_{i}\lambda \;\left(b_{i}\text{,}\;b_{i+1}\right)\text{,}$$

where *ω*_*i*_ is a weight factor which is dependent on the position of consecutive bases along the oligonucleotides, *b*_*i*_ is the *i*-th nucleotide of probe sequence, and λ is the stacking energy of the pair of nearest-neighbors along the probe. With λ(*b*_*i*_*b*_*i* + 1_) and *ω*_*i*_ known as basic constants which hardly change between arrays [39], *N* can be easily estimated by regression.

However, the model ignores cross-hybridization. There are two alleles (allele A and allele B) in the genome for a certain single polymorphic locus. For high sequence similarity, each allele has a high possibility of binding to the probe which is designed to interrogate the other allele. This cross-hybridization may bring bias when estimating the AC of target sequences (See [20] and Additional file 1). Therefore, we go one step further to improve the model by assuming that *I*_*s*_ follows an additive model of *I*_*sA*_ and *I*_*sB*_. Their meanings are clear: the contribution of allele A and B target sequences, respectively, to probe intensity. Both *I*_*sA*_ and *I*_*sB*_ can be modeled by Equation (2); thus our proposed model is

(4) $$I=\frac{N_{A}}{1+\exp\left(E_{A}\right)}+\frac{N_{B}}{1+\exp\left(E_{B}\right)}+I_{\mathrm{bg}}+\varepsilon \text{,}$$

where *N*_*A*_ and *N*_*B*_ are ACs for allele A and B, respectively, and *E*_*A*_ and *E*_*B*_ denote binding free energy. With quite a few probes in one probeset, the ordinary least squares (OLS) method yields unbiased estimates of *N*_*A*_ and *N*_*B*_. The summation of *N*_*A*_ and *N*_*B*_ gives the total concentration *N* (See [20] and Additional file 1). For the nonpolymorphic probe with only one allele, *N* can be straightforwardly obtained from Equation (2).

#### Normalization between arrays

In order to eliminate the systematic bias between arrays which may arise from the different library preparation conditions of the experimental process, we use the following transformation:

(5) $$N_{\mathrm{mk}}^{'}=N_{\mathrm{mk}}.\alpha_{m}\text{,}$$

where *N*_*mk*_ is the total concentration for array *m* at locus *k*, and $\alpha_{m}=\;2/median\left(N_{\mathrm{mk}}\text{,}k=\;1,\;2,\ldots ,K\right)$ is the normalization factor for array *m* (*K* = the total number of loci from one array).

#### Calibration for amplification efficiency

We have found that $N_{\mathrm{mk}}^{'}$ are fairly stable from one array to another, except for CNV regions for one certain locus *k* (see Additional file 1); therefore, a simple adjustment approach is proposed to calibrate the various amplification efficiencies:

(6) $${\hat{N}}_{\mathrm{mk}}=N_{\mathrm{mk}}^{'}\cdot \gamma k\text{,}$$

where $\gamma_{k}=2/median\;\left(N_{\mathrm{mk}}^{'}\text{,}\;m=1\text{,}\;2\text{,}\ldots \text{,}\;M\right)$is the adjustment factor for each locus *k* (*M* is the total number of reference samples). In order to estimate the adjustment factor $\gamma_{k}$_,_ a pool of reference samples is needed. In the case–control assay pattern, the control arrays are treated as the reference pool. In this article, the HapMap samples from dataset I are used to estimate $\gamma_{k}$. CNVhac takes ${\hat{N}}_{\mathrm{mk}}$ as the estimated raw CN for locus *k* in array *m*.

#### CNV calling

CNVhac implements a HMM-based algorithm to call CNVs. HMM methods have previously been successfully applied to other studies [13,41,42], and the main idea of our algorithm is similar to them. In our implementation of the HMM, the hidden state is the true CN ({0, 1, 2, 3 or >=4}) of each locus along the genome, and the observed state is our estimated raw CN ${\hat{N}}_{\mathrm{mk}}$. For each locus, the emission probabilities are estimated from a normal distribution with true CN as mean. The transition probability of jumping out from normal state is presumed to be low, whereas jumping back to a normal CN or transitioning within the same state is relatively high. Furthermore, the distance between neighboring loci is correlated with transition probability [13]. Given the initial emission and transition probabilities, the Viterbi algorithm [43] is used to decode the hidden states. Then, the parameters can be updated iteratively until converging. A more detailed description of this method can be found in Additional file 1.

## Results

The pipeline of CNVhac mainly consists of two major steps. The preprocessing step first estimates the raw CNs ${\hat{N}}_{\mathrm{mk}}$, and, second, the CNV calling step then searches for breakpoints through a HMM model. In this section, we compare CNVhac with two widely used raw CN estimation methods, CRMA_v2 (‘Copy-number estimation using Robust Multichip Analysis’ [6]) and cn.FARMS (‘factor analysis for robust microarray summarization’ [7]), to evaluate the accuracy of estimated raw CN ${\hat{N}}_{\mathrm{mk}}$. CRMA_v2 is an extension of CRMA [44] for estimating raw CNs for downstream analyses. cn.FARMS presents a probabilistic latent variable model for summarizing probes to obtain raw CN estimates. Both CRMA_v2 and cn.FARMS outperform other studies on raw CN estimation [6,7]. Meanwhile, to assess the performance of CNV calling, we compare CNVhac with another popular approach known as Birdsuite [13], which is asserted to be the best for CNV inference with Affymetrix SNP arrays [5]. Because Birdsuite does not estimate raw CNs, it is not considered in the comparison on raw CN estimation.

### Raw CN estimation on HapMap CEU samples

**Figure 1:**
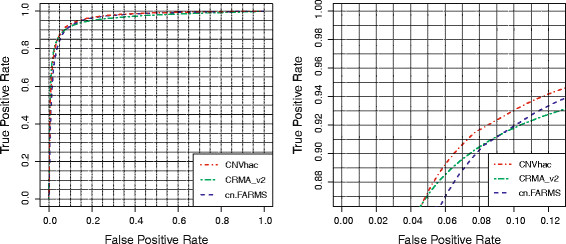
**ROC curves of the sex classification for CNVhac, CRMA_v2 and cn.FARMS on 59 HapMap CEU founders.** Left: Full ROC curves. Right: Top-left corner of ROC curves. CNVhac performs better than CRMA_v2 and cn.FARMS.

We assess the performance of raw CN estimation from two aspects: the accuracy in classifying the sex of HapMap individuals and the amplitude of genomic waviness. Females have two copies of X chromosome, while males only one; therefore, the CN of X chromosome can naturally be used as the benchmark to evaluate the power of the raw CN estimates to differentiate between one or two copies. We collected the same 59 CEU parents in Dataset I to do this classification task as [7]. Children were excluded to avoid inherited biases. The sample of female founder NA12145 was also removed on the basis of its low true CN level [44]. All the loci in the pseudoautosomal regions (PAR1 and PAR2), segmental duplications (http://humanparalogy.gs.washington.edu/build36) and CNV regions [38] in chromosome X were excluded owing to CN contamination. Finally, 83121 polymorphic and nonpolymorphic loci were kept which gives 4904139 (=83121 × 59) single locus classification tasks. The receiver operating characteristic (ROC) curve is introduced to assess the performance of different methods. The horizontal axis of the ROC curve represents the false positive rate (the fraction of males classified as females), while the vertical axis stands for the true positive rate (the fraction of females classified as females). Figure 1 shows the ROC for CNVhac, CRMA_v2 and cn.FARMS, respectively. The areas under ROC curve (AUCs) of CNVhac, CRMA_v2 and cn.FARMS are 0.9684, 0.9603 and 0.9627, respectively. We see that CNVhac outperforms CRMA_v2 and cn.FARMS when distinguishing males from females based on the estimated raw CNs.

**Figure 2:**
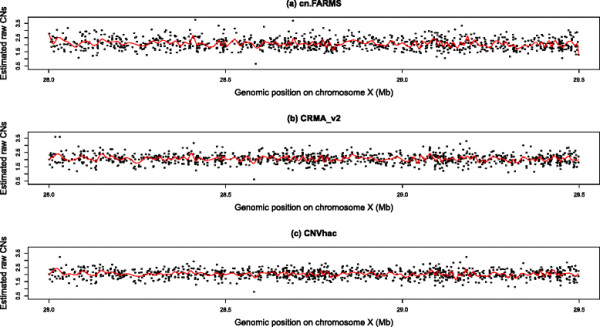
**Genomic wave patterns on a segment of Chromosome X of one CEU female founder, NA06985, for (a) cn.FARMS, (b) CRMA_v2 and (c) CNVhac.** CNVhac has the smallest amplitude of estimated raw CNs.

**Figure 3:**
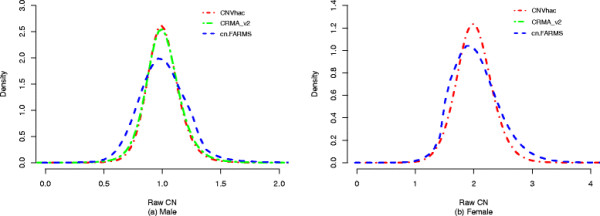
**Density of raw CNs estimated by different methods for (a) male CEU founders and (b) female CEU founders on chromosome X.** Raw CNs are scaled to the same median (for males 1 and females 2). CNVhac shows significantly smaller variance than CRMA_v2 and cn.FARMS (F test, all *p*-values are < 2e-16).

The better result of sex classification by CNVhac may be attributed to better control of genomic waviness. To assess the waviness, we investigated the estimated raw CNs of chromosome X used above. The three sets of raw CNs were separately scaled to the same median. For females, the median is set as 2 and for males 1. Figure 2 shows an example of dissimilar genomic wave patterns for one female CEU founder, NA06985. The fluctuation of raw CNs is obvious in cn.FARMS, with somewhat less fluctuation in CRMA_v2. However, the waves are smoothed most effectively by CNVhac compared to the other methods. Figure 3 shows the density of raw CNs for female CEU founders and male founders, respectively. More precisely, we computed the variance of raw CNs. For females, the variances of cn.FARMS, CRMA_v2 and CNVhac are 0.2118, 0.1225 and 0.1112. For males, the variances are 0.2597, 0.0336 and 0.0289. For both females and males, CNVhac has the smallest variance (F test, all *p*-values are < 2e-16). This result implies that CNVhac can smooth the fluctuation through one simple, but effective, method.

### CNV calling on HapMap samples

**Figure 4:**
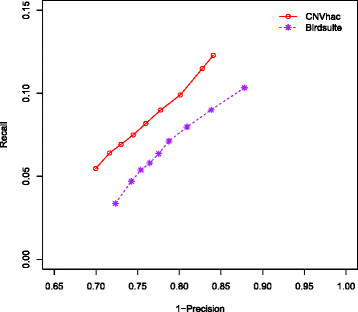
**1-precision versus recall curves for CNV detection on 269 HapMap samples.** A curve that is located more toward the upper-left corner indicates better performance. Note: FDR is 1-precision. Compared to Birdsuite, CNVhac shows an appreciably lower FDR when calling CNVs.

The cross-platform verified regions in dataset II are defined as true CNVs to assess the power of CNV detection for CNVhac and Birdsuite on the 269 samples from dataset I (NA19012 is missing in the result of [38]). We filtered out those verified regions having fewer than 5 probes designed in Affymertix SNP 6.0 array, resulting in 1381 verified regions for our evaluation. Each sample has a different number of CNVs annotated in the 1381 selected regions [38]. In total, we have 49662 true CNVs annotated in the 1381 regions across the 269 samples. We assessed the performance of each algorithm by calculating the ratio of the predicted CNVs, which are supported by true CNVs to all the predicted CNVs along the genome (precision), and the fraction of true CNVs, which are predicted by this algorithm (recall). The concordance principle for predicted and true CNVs is that more than 50% of either region is covered by the other. When calculating the precision and recall, we summed up all 269 samples. Through the default parameter settings, the precision and recall of Birdsuite are 40.01% (19337/48333) and 38.94% (19337/49662), while the counterparts of CNVhac are 43.45% (5828/13412) and 11.74% (5828/49662). Compared to Birdsuite, CNVhac has a higher precision, but a lower recall. Note that the results of Birdsuite contain a set of predefined common CNVs provided by another study [45], whereas CNVhac identifies CNVs without a source of predefined common CNVs. In GWAS analyses, false discoveries are inclined to occur when identifying rare CNVs [7]. Therefore, in the assessment of CNV calling power here, we removed the predefined common CNVs [45] from both the predicted and true CNVs. Altogether we have 22043 true CNVs across the 269 samples this time. The 1-precision versus recall curve which is similar to ROC is introduced to show the performance. A curve more in the upper-left corner indicates better performance. Figure 4 shows the 1-precision versus recall curve of CNV calling for all 269 HapMap samples in Dataset I. At comparable levels of recall, we see that CNVhac gives higher precision than Birdsuite. A higher precision means a lower false discovery rate (FDR). The result implies that our method calls CNVs with a lower FDR.

### Sample batch dependence of CNV calling

As described in the Background section, different parameters trained from different sample batches may cause an in-consistent inference. To evaluate the sample batch dependence of CNV calling of CNVhac, we compare it with Bird-suite. In CNVhac, estimating adjustment factor $\gamma_{k}$ is the only step requiring a batch of samples. In Section 3.2, all 270 HapMap samples were used to estimate $\gamma_{k}$. Here, we divided the 270 samples into 3 groups and then treated them as different pools of reference samples. Each group consisted of 90 samples. (The different choice of samples in each group can be found in Additional file 2). Adjustment factor $\gamma_{k}$ can be estimated within each group, respectively. With the different $\gamma_{k}$, raw CN estimates ${\hat{N}}_{\mathrm{mk}}$ change, as well as the CNV calling. For a specific sample *S*_*i*_, three sets of CNV regions can be detected through different $\gamma_{k}$. We assess the batch dependence by computing the ratio of intersection regions to union. For Birdsuite, 3 groups were created by the same way. Next, sample *S*_*i*_ was put to the other two groups which do not contain it. Hence, one can also obtain three sets of identified CNVs. We chose 6 individuals (2 CEU, 2 YRI, 1JPT and 1CHB) to call CNVs based on different groups. Table 1 displays the ratio of intersection to union, respectively, under default parameter setting. From this, we see that CNVhac shows significantly higher ratios than Birdsuite (*p*-value = 6.5e-3 by Wilcoxon rank-sum test). This indicates that CNVhac alleviates the sample batch dependence of CNV calling to a certain extent.

**Table 1:** Results of CNV calling based on different training sample batches for CNVhac and Birdsuite

	**Birdsuite**						**CNVhac**					
	**G1**^**§**^	**G2**	**G3**	**I**^**¶**^	**U**^**†**^	**Ratio**^**‡**^	**G1**	**G2**	**G3**	**I**	**U**	**Ratio**
NA12156	17	19	21	14	22	0.64	15	17	18	15	17	0.88
NA12878	22	21	19	15	28	0.54	29	26	24	20	33	0.61
NA18507	19	15	20	10	23	0.43	16	20	20	15	21	0.71
NA18517	20	21	21	14	25	0.56	21	21	18	16	23	0.7
NA18555	16	16	15	11	20	0.55	16	14	17	11	18	0.61
NA18956	13	12	16	9	16	0.6	20	21	24	16	24	0.67

§The number of predicted CNVs using group 1 for parameter training.

^¶^The number of CNVs in intersection set of “G1”, “G2” and “G3”.

^†^The number of CNVs in union set of “G1”, “G2” and “G3”.

^‡^The ratio of intersection to union.

## Discussion

For years, the array-based technologies have been widely used for exploring CNV events. However, the inherent noise of microarray data may lead to high FDR when making inferences. In array experiments, hybridization is highly correlated with the sequence constitutions [27,28,30,32,39,40,46]. The binding affinities of probes can be subject to large variability by the various sequences. Most previous algorithms attempt to model the binding affinity through statistical or empirical methods [41,44], which need multiple samples for training parameters. However, such multiple samples may lead to another problem: sample dependence of outputs [26]. The various choices of training samples may result in different estimated parameters, leading, in turn, to incompatible results. All the algorithms which need multiple training samples have a possibility encountering this effect. Consequently, strategies based on single-array processing are preferred. Up to now, however, few single-array approaches have been presented. CRMA_v2 is a single-array preprocessing method for SNP array analysis. However, the raw CNs estimated by CRMA_v2 exhibit a wavy pattern, and thus may not be accurate enough for downstream CNV identification.

Motivated by addressing the cross-hybridization of probes, genomic waves of intensities and sample dependence of parameter estimation, we propose in this article a single-array preprocessing method, termed CNVhac, to estimate more accurate raw CNs. Based on the previous PICR method [20], we model the hybridization and cross-hybridization of probes through physicochemical law. Wan et al. have shown that the PICR model can address the cross-hybridization effect very well [20]. The genomic wave patterns of signal intensities are hypothesized to reflect the various amplification efficiencies of DNA fragments in the PCR process [33]. However, based on the diversity of sheared fragments and complicated PCR procedures, it is difficult to estimate the accurate amplification rate for each locus. Instead, we smooth the genomic waves by estimating an adjustment factor for each locus since we have found that the estimated CNs show a fairly stable pattern between loci (see Additional file 1). Compared to CRMA_v2 and cn.FARMS, this simple calibration method effectively reduces the amplitude of waviness. Note that the reduction of waviness is not simply a compression of variance in that CNVhac provides more accurate raw CN estimates which can well differentiate between one or two copies. Moreover, the number of parameters needed to estimate target concentration ${\hat{N}}_{\mathrm{mk}}$in CNVhac is much fewer than prior statistical models and can be estimated from one single array quite stably [39]. This property avoids the sample dependence of parameter estimation. Compared to one popular CNV detection method known as Birdsuite [5,13], CNVhac, indeed, alleviates the sample dependence of CNV calling more effectively. However, CNVhac needs a pool of reference samples to estimate $\gamma_{k}$ for calibrating amplification efficiency. In the case–control assay pattern, the control samples are treated as the reference pool. While the dataset contains only case samples, anonymous normal samples, e.g., HapMap samples, can be used as the reference pool. Because of the different experimental conditions, the anonymous normal samples may bring sample-dependent bias for $\gamma_{k}$. Actually, CNVhac cannot address this kind of sample dependence.

CNVs have attracted much attention in recent years because they are assumed to play a significant role in causing human disease [1,4]. Especially, some recent studies and reviews have shown that rare CNVs contribute much more to neuropsychiatric disorders than previously thought [2,47-51]. However, the mechanism underlying the influence of CNVs on human phenotypes is still not well understood. Furthermore, even a small fraction of false discoveries may introduce misunderstanding in the downstream association studies. Therefore, CNV calling methods are strongly de-sired to control the FDR [7]. On the basis of raw CN estimates with cross-hybridization and amplification rate correction, CNVhac can identify rare CNVs with a lower FDR compared to the powerful Birdsuite method. This result implies that CNVhac can accurately identify CNVs, especially rare CNVs, for downstream association studies.

Since CNVhac is a single-array based strategy, the running time could be reduced by executing CNVhac on multiple processors in parallel when analyzing a large set of samples. Also, since parameters are consistent between arrays, there is no need to reprocess the early data when new samples are hybridized.

## Conclusion

Cross-hybridization and different amplification efficiencies of probes are the common difficulties in microarray analysis. Most studies attempt to solve the problem by training numerous model parameters from a large dataset, but this might incur inconsistent results. Moreover, the statistical power of this methodology may be significantly reduced when the training dataset is not big enough. In this article, we first addressed cross-hybridization problem through physico-chemical law and then proposed a simple adjustment for the various amplification rates. Our method, CNVhac, avoids complicated statistical models which need many samples for training. By comparing CNVhac with other methods, we have established that our simple process is effective and suitable for all Affymetrix SNP array types with similar design standards. Finally, the working principle of CNVhac can be easily extended to other platforms, such as Illumina and Agilent arrays.

## Endnotes

CNVhac^a^: The algorithm is implemented in R and C++ and is available at http://www.math.pku.edu.cn/teachers/dengmh/CNVhac.

## Abbreviations

CN, Copy number; CNV, Copy number variation; FDR, False discovery rate; AC, Allelic concentration; HMM, Hidden Markov Model; GWAS, Genome-wide association studies; PICR, Probe intensity composite representation; PDNN, Position-dependent nearest-neighbor; OLS, Ordinary least squares; CRMA, Copy-number estimation using Robust Multichip Analysis; cn.FARMS, Factor analysis for robust microarray summarization; ROC, Receiver operating characteristic; AUC, Area under ROC curve.

## Competing interests

The authors declare that they have no competing interests.

## Authors’ contributions

MPQ and MHD conceived the project. MPQ, LW and MHD proposed the main idea. QW and PCP developed the program. QW implemented the methods, analyzed the data, and wrote the manuscript. MPQ, LW and MHD finalized the manuscript. All authors read and approved the final manuscript.

## Funding

This work was supported by the National Natural Science Foundation of China [No.31171262, No.11021463] and the National Key Basic Research Project of China [No.2009CB918503].

## Pre-publication history

The pre-publication history for this paper can be accessed here:

http://www.biomedcentral.com/1755-8794/5/24/prepub
